# Increasing Serious Illness Conversations in Patients at High Risk of One-Year Mortality Using Improvement Science: A Quality Improvement Study

**DOI:** 10.3390/healthcare13020199

**Published:** 2025-01-20

**Authors:** Kanishk D. Sharma, Sandip A. Godambe, Prachi P. Chavan, Agatha Parks-Savage, Marissa Galicia-Castillo

**Affiliations:** 1 Division of Geriatric Medicine, Donald and Barbara Zucker School of Medicine at Hofstra/Northwell, Hempstead, NY 11549, USA; 2 Department of Pediatrics and Emergency Medicine, University of California at Irvine, Irvine, California and Children’s Hospital of Orange County, Orange, CA 92868, USA; sgodambe@choc.org; 3 Master of Public Health Program, School of Health Professions, Macon & Joan Brock Virginia Health Sciences at Old Dominion University, Norfolk, VA 23508, USA; chavanpp@odu.edu; 4 Department of Family & Community Medicine, Graduate Medical Education, Eastern Virginia Medical School at Old Dominion University, Norfolk, VA 23508, USA; parksac@odu.edu; 5 Department of Medicine, Division of Geriatrics and Palliative Medicine, Glennan Center for Geriatrics and Gerontology, Eastern Virginia Medical School at Old Dominion University, Norfolk, VA 23508, USA; galicimc@evms.edu

**Keywords:** prognostication tool, serious illness conversation, quality improvement, advanced care planning

## Abstract

**Background:** Serious illness conversation (SIC) in an important skillset for clinicians. A review of mortality meetings from an urban academic hospital highlighted the need for early engagement in SICs and advance care planning (ACP) to align medical treatments with patient-centered outcomes. The aim of this study was to increase SICs and their documentation in patients with low one-year survival probability identified by updated Charlson Comorbidity Index (CCI) scores. **Methods:** This was a quality improvement study with data collected pre- and post-intervention at a large urban level one trauma center in Virginia, which also serves as a primary teaching hospital to about 400 residents and fellows. Patient chart reviews were completed to assess medical records and hospitalization data. Chi square tests were used to identify statistical significance with the alpha level set at <0.05. Integrated care managers were trained to identify and discuss high CCI scores during interdisciplinary rounds. Providers were encouraged to document SICs with identified patients in extent of care (EOC) notes within the hospital’s cloud-based electronic health record known as EPIC. **Results:** Sixty-two patients with high CCI scores were documented, with 16 (25.81%, *p* = 0.0001) having EOC notes. Patients with documented EOC notes were significantly more likely to change their focus of care, prompting palliative care (63.04% vs. 50%, *p* = 0.007) and hospice consults (93.48% vs. 68.75%, *p* = 0.01), compared to those without. Post-intervention surveys revealed that although 50% of providers conducted SICs, fewer used EOC notes for documentation. **Conclusion:** This initial intervention suggests that the documentation of SICs increases engagement in ACP, palliative care, hospice consultations, and do not resuscitate decisions.

## 1. Introduction

Conversations between patients, caregivers, and clinicians about patients’ preferences, values, and goals of care can be termed as serious illness conversations (SICs) [1]. These conversations can increase the quality of life for patients and reduce healthcare costs, as well reducing aggressive interventions at end-of-life [2,3,4]. SICs aim to align medical care and treatment with patients’ values, goals, priorities, and preferences [5]. During the COVID-19 pandemic, the importance of early SICs resurfaced as the need for rapid decision-making with deteriorating patients became evident [6]. Therefore, it is crucial for physicians to identify patients at high risk of one-year mortality to conduct early and frequent SICs.

Physicians still often delay SICs until late in a patient’s disease course. One study showed that three-fourths of patients with renal disease who died in acute care facilities were not enrolled in hospice, signifying the further importance of SICs [7]. Another study showed that out of 584 patients with chronic kidney disease, less than 10% had end-of-life care discussions with their nephrologist in the previous 12 months [8]. Physicians are generally overoptimistic about patient outcomes, often inaccurately estimating the survival times for seriously ill patients [9].

Various interventions have been implemented in hospital settings to increase the frequency and quality of SICs. Nursing-led interventions based on an established SIC guide have been effective in increasing the documentation of these conversations [10]. A randomized control trial showed that clinician-facing communication priming interventions can significantly improve the documentation of SICs in electronic health records (EHRs) [11]. Other studies have used EHR alerts to trigger SICs but these did not use validated mortality prediction tools [12].

In 2011, Quan et al. published an updated scoring system for the Charlson Comorbidity Index (CCI), a method of predicting the one-year mortality risk by classifying comorbid conditions [13].

The Sentara Health System integrated this tool into the EHR. The CCI score is calculated based on each patient’s problem list and provides a predictive percentage score for one-year survival. This score appears in a column in EPIC’s patient list and is color-coded with 0–53% as red, 54–77% as yellow, and 78–100% as green. The study aimed to use the CCI score to trigger SICs in patients with the highest risk of predicted mortality.

## 2. Materials and Methods

### 2.1. Setting and Participants

This study was conducted at a large urban hospital, the primary teaching hospital, associated with a medical school. Both the academic group and private hospitalist groups were involved in the project. There were five resident physician teams under each academic group, consisting of two interns and one senior resident physician. It is important to note that this paper sometimes refers to the academic group and hospitalist group as “providers” for ease of use.

### 2.2. Study Design

The hospital mortality review committee meets regularly to discuss patient deaths to identify preventable causes and areas for improving the quality of care. A prominent concern was the low frequency or late timing of SICs among seriously ill patients. This led to the formation of a quality improvement (QI) team that included an academic hospitalist, QI coordinators, and a geriatrician. The team followed the Model for Improvement Guidelines to lead this initiative as a QI study [14].

### 2.3. Cause-and-Effect Analysis and Driver Diagram

**Figure 1:**
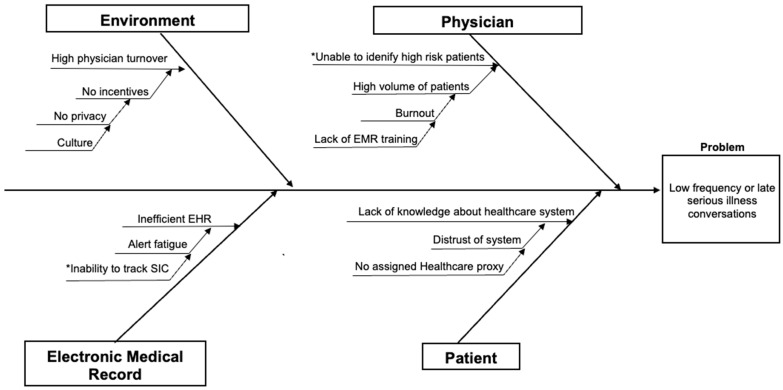
Cause-and-effect diagram. “*” Items were prioritized during data collection.

A cause-and-effect diagram (Figure 1) was created to assess the reasons for the low frequency of SICs in four broad categories: environment, physician, EHR, and patient. Within the environmental category, the QI team identified three important causes for the low frequency or absence of SICs: (i) the lack of private spaces for these crucial conversations; (ii) the lack of resident physicians and hospitalists’ continuity; (iii) an organizational culture that did not prioritize these conversations. Other potential factors included the inability to identify patients at high risk of one-year mortality, lack of time due to high patient volume, physician burnout, and inadequate EHR training for documenting SICs.

**Figure 2:**
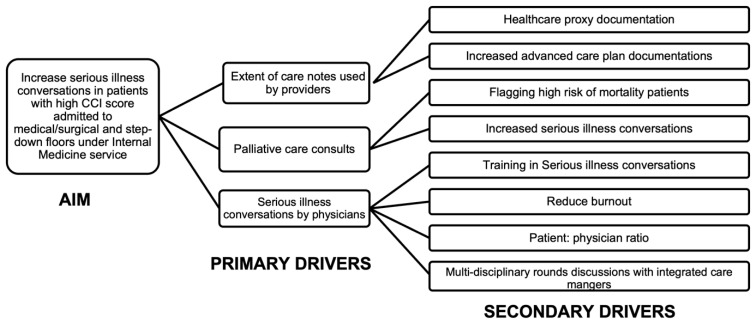
Driver diagram. Abbreviations: CCI = Charlson Comorbidity Index.

In the EHR category, it was noted that inefficient EHR systems can hinder SIC documentation, multiple alerts may divert attention from high-risk patients, and the EHR may not efficiently track SICs. Patient-related factors include a missing medical power of attorney, distrust in the system, or insufficient healthcare knowledge to make informed decisions from SICs. Based on the team’s prioritization and discussions, a driver diagram (Figure 2) was formulated to understand the factors driving the goal of increasing SICs. The figure depicts the primary drivers of this study’s objective, which were influenced by secondary factors. The intervention of this study focused on identifying high-risk one-year mortality patients to improve SIC documentation and increase palliative medicine consults.

With the integration of the CCI into the EHR, identifying high-risk patients for one-year mortality became feasible. The QI team recognized that this identification can lead to earlier and more frequent SICs.

### 2.4. Intervention

For this article, only one plan–do–study–act (PDSA) cycle was done. During the “plan” phase of the PDSA cycle, the utility of the CCI prognostication tool was discussed with the providers and integrated care managers. The prior workflow did not include a CCI score column in the daily generated patient list for integrated care managers. The score was incorporated into each provider and integrated care manager’s patient list. This was to start discussions during multi-disciplinary rounds to determine if patients with high CCI scores had a SIC with their providers. If the SIC was complex, a palliative medicine consultation was obtained.

**Figure 3:**
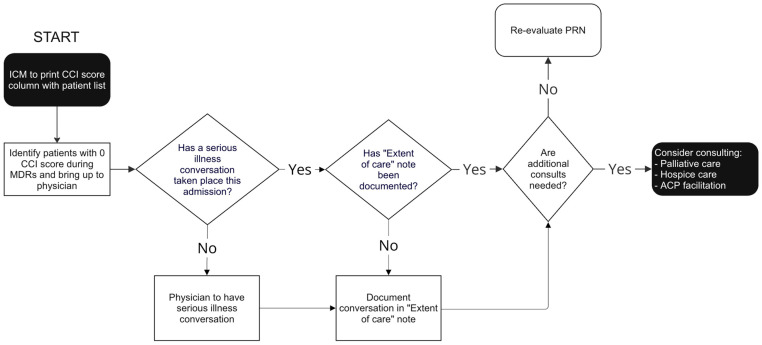
Map of new process to increase serious illness conversations. ICM: Integrated Care Manager; CCI: Charlson Comorbidity Index; ACP: Advance Care Planning.

The QI team mapped out a new workflow to increase SICs, identifying each step and providing a clear visual guide for the staff involved, as depicted in the process map (Figure 3). Physicians were encouraged to document each SIC in an EOC note. The workflow required minimal changes and empowered providers with evidence-based data to identify patients at high risk of one-year mortality.

The QI coordinators performed random audits during multidisciplinary rounds to observe workflows and address questions. A one-page educational summary was developed for physicians explaining the CCI score and its use in identifying patients who would benefit from a SIC. These handouts were distributed during education sessions and rounds.

### 2.5. Inclusion and Exclusion Criteria

The initiative was piloted in a 30-bed medical–surgical unit and 24-bed step-down unit of the hospital from September 2022 to December 2022. All patients admitted to these units identified by the CCI tool as having a 0% chance of one-year survival were eligible to be part of the study. A daily census on average had 18% patients with a 0% chance of survival on the 2 units. Any patients with higher chances of survival as per the CCI tool were excluded. The data collected for all patients were de-identified for analysis.

### 2.6. Data Management

The following variables were collected through a manual chart review: patient name, medical record number, change in code status, presence of ACP documents on file, EOC note documentation, outcome of SIC, palliative medicine consult, number of hospitalizations in the past two years, hospice consult, and dates of admission and discharge. The data were stored in a password-protected Excel file. A retrospective chart review was conducted two weeks after the initial multi-disciplinary rounds discussion to update the initial data.

### 2.7. Qualitative Feedback and Stakeholder Engagement

The team met monthly with hospitalists through integrated care manager meetings and internal medicine resident physician forums. All data and related analyses were shared with the stakeholders to gather qualitative feedback that would help understand how the providers felt about new workflow. A questionnaire for qualitative feedback was created using the secure web application for managing online surveys called RedCap and distributed via email.

### 2.8. Measures

This study’s outcome and process measures are shown in Table 1.

At the end of the third month, the QI team initiated the “study” phase of the PDSA cycle, where the data compilation and analysis were completed.

### 2.9. Statistical Analysis

The data collected were analyzed by comparing the EOC note variable (the dependent variable) with the independent variables in the study, namely the frequency of having ACP on file, palliative medicine consults, hospice consults, and the do not resuscitate (DNR) code status. The categorical variables were analyzed using frequencies and percentages, while the continuous variables were analyzed using the mean and standard deviation. Fisher’s exact chi-square test was used for variables where the cell count was <5, and Pearson’s chi-square was used for all other variables. The *p*-value was set at <0.05 for a two-sided test. All analyses were performed using SAS statistical software version 9.4.

### 2.10. Ethics

This project was submitted for evaluation by the Institutional Review Board (IRB) of Eastern Virginia Medical School and was deemed exempt from IRB review. Participation in the RedCap survey was voluntary, and the responses were anonymous.

## 3. Results

A chart review revealed that out of these 62 patients, 16 had extent of care (EOC) notes documented. Due to a staffing shortage, data collection from the step-down floor could not be completed.

Table 2 shows the association of EOC notes with other outcome variables. Of the 62 patients, EOC notes were present for 16 patients (25.81%, *p* = 0.0001). The patients with EOC notes were more likely to have ACP documentation on file (*n* = 10, 62.50%, *p* = 0.01). Additionally, 81% (*n* = 13) of the patients with EOC notes had a DNR status, while only 18% (n = 3) had a full code status. Furthermore, 50% (*n* = 8) of those with EOC notes opted for palliative care consults, whereas 31.25% (*n* = 5) had hospice consults documented. All values, except for the DNR code status, were found to be statistically significant (*p* < 0.05).

### Post-Intervention Feedback Using Redcap Survey

**Figure 4:**
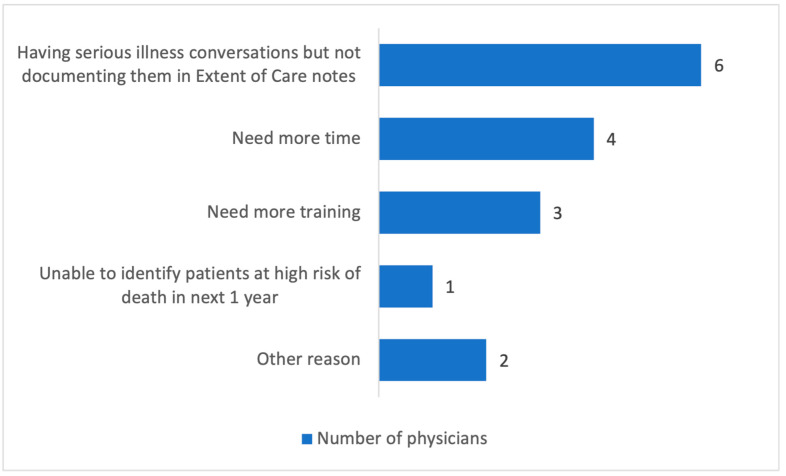
Graph depicting what barriers were felt by physicians in having serious illness conversations with patients.

Monthly meetings were held with resident physicians and hospitalists to share results and gather feedback. During the presentations, 8 out of 12 resident physicians and 4 out of 8 hospitalists responded to the survey. Three of the 12 providers reported using the CCI tool in the past month, and 6 of the 12 providers used EOC notes to document SICs with patients. Figure 4 illustrates the barriers physicians felt in having SICs with patients.

## 4. Discussion

This initiative demonstrated that using a prognostication tool to identify patients with a high risk of one-year mortality effectively triggered SICs. The patients with documented SICs showed increased frequencies of ACP documentation, palliative medicine consults, hospice consults, and DNR code statuses. The use of EOC notes for documenting SICs was likely facilitated by reminders from integrated care managers during multi-disciplinary rounds. This study’s findings suggest that timely SICs can encourage changes in code status, initiate palliative medicine, and hospice consultations.

An outpatient study to increase SIC documentation among patients with advanced cancer achieved a 30–40% documentation range in one year [15]. In comparison to the outpatient study, this study’s data showed that 26% of patients had documented SICs.

Other interventions to trigger SICs include asking the “surprise” question, “Would I be surprised if this person dies in the next year?” One study demonstrated a seven-fold increase in the likelihood of death if physicians answered “no” to this question [16]. However, since physicians tend to overestimate patients’ life expectancy, supplementing the surprise question with objective clinical criteria has been suggested [17,18]. This study‘s intervention used an EHR-generated CCI score as an objective clinical criterion.

Similar tools have been used to trigger SICs, such as the E-Gagne tool, which identified patients at an elevated risk of mortality within one year. A study demonstrated increased SIC documentation from 0 to 88% using this tool [19].

A study from Haley et al. showed that interventions increased SICs from 20.5% for the control group compared to 44.6% in the intervention group. The intervention included a ten-minute didactic session and electronic reminders for SICs [20].

### 4.1. Strengths

This study’s strengths include the meticulous planning before initiating the intervention. The QI committee identified and included all relevant stakeholders, such as the integrated care manager director, QI coordinators, internal medicine, and geriatrics physicians. Collecting both quantitative and qualitative data provided valuable feedback from providers, offering grassroots-level input for future PDSA cycles. Additionally, the ease of implementing the intervention was a strength, as discussing prognostic indicators during the multi-disciplinary rounds required minimal time for each patient. Furthermore, the real-time calculation and updating of the CCI prognosis by the EPIC EHR system enabled continuous discussions about patient prognoses throughout hospitalization.

### 4.2. Limitations

The first limitation of this study was that not all high-risk patients’ data were captured during the multi-disciplinary rounds. The integrated care manager limited the data collection as time permitted during these rounds, and the lack of a permanent care manager resulted in missing patient data from the step-down floor unit. In the future, interventions can be tried on a smaller scale restricted to one unit and learning from the flow of the process to make improvements.

The second limitation was the lack of continuity of care from the integrated care managers during the intervention weeks due to increased workload, lack of appropriate staffing, and increased staff turnover. This could again be mitigated by focusing on a single unit or floor that has stable staffing.

The third barrier was informing all hospitalists and resident physicians about the intervention and the use of the CCI tool, as not all group members attended the monthly meetings and were, therefore, unaware of the prognostication tool.

Fourth, there was no established baseline for the percentage of patients with documented SICs before starting the intervention, as these data were not readily available and required manual collection during the multi-disciplinary rounds. To prevent this in the future, the QI team could review the charts of high-risk patients on pre-specified days before the intervention.

The use of a prognostic tool, such as the CCI score, during multi-disciplinary rounds can effectively identify patients at the highest risk of one-year mortality. This early identification can prompt timely SICs and improve the accuracy of physicians’ prognostications [9]. The SICs are crucial for aligning medical care with patients’ goals and values, especially for those at high risk of mortality. Prognostic tools such as the CCI score help eliminate subjective biases and ensure equal prognostication opportunities for all patients, promoting more objective and consistent care.

### 4.3. Future Direction

Given the positive results from the first PDSA cycle, the authors anticipate that subsequent cycles will further enhance the utilization of the CCI tool and the documentation of SICs. The QI team will learn from the current limitations and qualitative feedback to incorporate it into the next PDSA cycle. The team will also collect data related to a patient’s cultural background to uncover disparities. The ultimate goal is for physicians to incorporate the CCI score into their routine chart reviews independently, without needing prompts from integrated care managers during the multi-disciplinary rounds. Since the biggest barrier identified was providers not documenting SICs, sharing the first PDSA cycle data would encourage documentation. Future PDSA cycles could focus on secondary drivers, such as improving physician training on effective SICs and implementing interventions to reduce physician burnout.

This study’s intervention has inspired another initiative at the Heart Hospital, which uses the same electronic health record (EHR) system. This new initiative applies the CCI tool to patients admitted with heart conditions, including heart failure exacerbation, coronary artery disease, and cardiomyopathies, to trigger timely SICs and improve patient outcomes.

## 5. Conclusions

The implementation of the CCI tool, which predicts one-year mortality, encourages the documentation of SICs in the EHR during multi-disciplinary rounds. This leads to increased ACP documentation, palliative medicine consults, hospice consults, and DNR code statuses. This simple, efficient, and cost-effective intervention can be adopted by other hospitals and health systems using similar EHRs, promoting more goal-concordant care for patients at the highest risk of mortality.

## Figures and Tables

**Table 1:** Measures and corresponding operational definitions. Abbreviations: ACP = advance care plan; DNR = do not resuscitate.

Measure	Operational Definition
Patients with a high risk of one-year mortality (process measure)	Number of patients admitted under medicine service with 0% chance of survival in the next year as predicted by CCI tool. Referred to as patients having “high CCI scores”.
Documented EOC note (process measure)	Percentage of patients admitted under medicine service having EOC notes with documented SIC during active hospitalization (yes/no).
ACP document on file (outcome measure)	Percentage of patients admitted under medicine service having an ACP on file (yes/no).
Palliative care consult (outcome measure)	Percentage of patients admitted under medicine service having a documented palliative medicine note on file during active hospitalization (yes/no).
Hospice consult (outcome measure)	Percentage of patients admitted under medicine service visited by a hospice care liaison with a written note in the chart during active hospitalization (yes/no).
DNR code status (outcome measure)	Percentage of patients admitted under medicine service with current code status of DNR, irrespective of the timing of the change from complete code during any hospitalization (yes/no).

**Table 2:** Association of extent of care notes with other outcome variables. Abbreviations: EOC = extent of care; ACP = advanced care plan; DNR = do not resuscitate; DNI = do not intubate.

	EOC Note (N = 62)		*p*-Value
	Present (N, %)	Absent (N, %)	
Number of patients with	16 (25.81)	46 (74.19)	0.0001
ACP documentation			
Yes	10 (62.50)	13 (28.26)	0.01
No	6 (37.50)	33 (71.74)	
DNR code status			
DNR/DNI; DNR with limits	13 (81.25)	41 (89.13)	0.41
Full status/comfort care	3 (18.75)	5 (10.87)	
Palliative care consult			
Yes	8 (50)	7 (15.22)	0.005
No	8 (50)	39 (84.78)	
Hospice consult			
Yes	5 (31.25)	3 (6.52)	0.02
No	11 (68.75)	43 (93.48)	

## Data Availability

Patient data cannot be made available due to privacy concerns as they may contain patient health information.

## Abbreviations

The following abbreviations are used in this manuscript:

SIC	Serious illness conversation
QI	Quality improvement
ACP	Advance care plan
CCI	Charlson Comorbidity Index
EOC	Extent of care
EHR	Electronic health record
IRB	Institutional Review Board
PDSA	Plan–do–study–act
DNR	Do not resuscitate
DNI	Do not intubate
